# Psychometric properties and measurement invariance of the Vaccination Attitudes Examination Scale (VAX) in a Spanish sample

**DOI:** 10.1186/s40359-022-00929-y

**Published:** 2022-09-19

**Authors:** Begoña Espejo, Irene Checa, Marta Martín-Carbonell

**Affiliations:** 1 Department of Methodology of Behavioural Sciences, Faculty of Psychology, University of Valencia, Av. Blasco Ibáñez, 21, 46010 Valencia, Spain; 2 Cooperative University of Colombia, Santa Marta, Colombia

**Keywords:** Vaccine hesitancy, Vaccine reluctance, Confirmatory factor analysis, Structural equation modelling, Measurement invariance, Educational level, Gender, COVID-19

## Abstract

In these times of pandemic, the acceptance or rejection of vaccines has become increasingly clear, with a considerable rise in the anti-vaccine movement in Spain. It is important to understand the attitudes that lead a person to refuse vaccination in order to develop more effective public health campaigns. The objective of this study has been to study the psychometric properties and measurement invariance of the Vaccination Attitudes Examination (VAX) scale in a Spanish sample. Confirmatory factor analysis and structural equation modelling have been used to study the psychometric properties of the VAX. Likewise, the measurement invariance by gender and educational level has been studied. The structure of four related factors for VAX is confirmed, as well as its predictive value, since the factor “trust in the benefit of the vaccine” clearly predicts the choice to be vaccinated. The strong measurement invariance by gender and educational level is also confirmed. The comparison of latent means between groups indicates that there are no differences by gender in any factor. However, people with a high educational level present higher scores in factors “concern about unforeseen future effects”, “concern about commercial effects and speculation” and “preference for natural immunity”. The VAX is presented as a reliable and valid tool to assess four different factors related to attitudes towards vaccines in Spain. Future studies of its cross-cultural invariance may help to determine the main factors that lead people not to be vaccinated in order to develop more effective public health campaigns.

**Supplementary Information:**

The online version contains supplementary material available at 10.1186/s40359-022-00929-y.

## Introduction

Despite the proven efficacy of vaccines in reducing mortality and morbidity from vaccine-preventable diseases, vaccination rates have been declining for years in many areas of the world even before the coronavirus pandemic [1]. This has led to the resurgence of some diseases that were largely controlled or eradicated. Refusal to be vaccinated has been linked, in some countries, to outbreaks of pertussis, measles, and other vaccine-preventable diseases [2, 3]. Likewise, in a review carried out with data from 33 countries, it was observed that the Covid-19 vaccination acceptance rates worldwide were very different. In some countries, such as the United States, Russia, Poland, Italy and France, the acceptance rates were less than 60%, and in countries such as Kuwait or Jordan they were less than 30% [4]. In Spain, until May 2022, the percentage of people who have received the full schedule of the Covid-19 vaccine is 92.6% [5]. But vaccine hesitancy is an emerging public health problem in Spain, which is slowing down the process of eliminating measles and other diseases [6–8].

Although the decision to be vaccinated or not is individual, it is influenced by the historical, political and sociocultural context of the reference country where the vaccination is carried out [9]. The media, as well as social networks, have great influence on a significant part of the population. In a recent study carried out in Spain, the coverage that the printed media carried out on vaccines between 2012 and 2017. It was observed that the tone of most articles changed from negative in 2012 to positive and neutral until 2017. It was also found that the fewer articles with a negative tone, the higher the vaccination rates [10]. Another study also carried out in Spain analysed the influence of anti-vaccine groups through social networks. The objective was to study the ideology of these groups using discourse analysis, in order to prepare responses based on scientific evidence. The results showed that the speeches of these anti-vaccine groups refer to aspects related to the safety and effectiveness of vaccines. They also strongly emphasize the importance of people’s values and beliefs, and that each person should freely choose whether or not to vaccinate. Likewise, it was observed that the argument that stands out the most is the distrust in health personnel and in official sources of information, which are governments and pharmaceutical companies [11]. In a more recent study, the Covid-19 anti-vaccination messages published on Twitter during December 2020 were analysed to find the key elements in their communication strategy. The results again raised arguments about the safety of vaccines, including viewing the vaccine as a means of manipulating the human genetic code [12]. Therefore, it is important to understand the attitudes that lead a person to refuse vaccination in order to develop more effective public health campaigns.

Regarding the instruments used to assess attitudes towards vaccination, there are several scales designed to assess the attitudes of parents towards vaccinating their children. For example, the *Attitudes and Behaviours Regarding Vaccination Decisions* [13], the *Parent Attitudes about Childhood Vaccines Survey* [14] and the *Vaccine Hesitancy Scale* [15]. Recently, this scale has been modified to also assess the attitude of parents towards specific vaccines, such as influenza and human papillomavirus [16], and for the assessment of attitudes towards vaccination against coronavirus in people with acquired immunodeficiency virus (HIV) [17]. There are also other scales designed to assess attitudes towards specific vaccines in adults, such us the *HIV Vaccine Attitudes Scale* [18], a variation of the *Vaccination Hesitancy Scale* adapted to assess adult attitudes toward influenza and coronavirus vaccination in the United States and China [19], and more recently, the *COVID-Vaccination Attitude Scale* [20].

The only existing instrument used to assess attitudes toward vaccines, without focusing on a specific population or vaccine, is the *Vaccination Attitudes Examination* (VAX) scale [21]. Although attitudes towards vaccination may differ depending on the type of vaccine, recent studies have shown that the reasons are often similar, including being against vaccine, including coronavirus vaccines, lack of confidence, concerns about their safety, doubts about the origin of vaccines, and vaccines safety due to its rapid development [22, 23]. Therefore, a single measure may be the most efficient way to identify people with vaccine concerns.

VAX adaptations have been carried out in several countries. In the United Kingdom the psychometric properties of the original version of the scale have been studied, with good results [24]. It has also been adapted in Turkey [25] and Romania [26], and its psychometric properties have been studied in a Spanish sample [27], but of these three countries, only the Turkish version performs a back-translation of the items, as advised by the International Test Commission [28]. In all cases, construct validity was studied using Exploratory Factor Analysis (EFA) and/or Confirmatory Factor Analysis (CFA), obtaining the best solution with four related factors. In some countries, convergent validity has also been studied with measures of susceptibility to possible adverse effects of the medication, with current health or with medical mistrust. The predictive validity of the VAX has been studied in the United States and the United Kingdom asking the participants about their intention to vaccinate themselves or their children, if they had been vaccinated against the flu the previous year, or if they would be vaccinated next year.

Regarding the study carried out in Spain, in addition to not carrying out the back-translation, the analyses of the study lead to uncertain conclusions. In addition to performing an EFA that offers a one-factor solution, the authors perform a CFA to study whether the one-factor structure or the four-related factor structure is better. In addition, Cronbach’s alpha coefficient is offered, instead of the recommended McDonald’s omega coefficient [29] for rating scales, or the equivalent Composite Reliability index [30, 31]. These are the most appropriate reliability indicators when factor loadings of a CFA are used. Furthermore, after confirming that the four-factor model fits better, the authors carry out a convergent and concurrent validity study. For concurrent validity, measures similar to those of previous studies are used, and for concurrent validity, the intention to be vaccinated against COVID-19 when the vaccine is available is used. However, in both cases, Pearson correlations are calculated with the total VAX score, instead of using the total score in each of the four factors, which, given the results of the construct validity study, is inadequate.

On the other hand, measurement invariance has not been studied in any country, but it is important to check the existence of measurement invariance to be able to make comparisons between groups. Furthermore, in all convergent and concurrent validity analyses, correlations between total scores or regressions have been used. For all these reasons, the main objective of this study has been to adapt the Vaccination Examination Attitudes (VAX) scale in a Spanish sample, larger than those used in all previous studies, and study its psychometric properties (not only construct validity) and measurement invariance using structural equation modelling, which offers less measurement error than the calculation of Pearson correlations with total observed scores.

## Methods

### Procedure

Data was collected online between November 15, 2021 and March 7, 2022. It was used the LimeSurvey platform, installed on the University’s servers, which allows us to guarantee the protection of the data by our university. The survey was completely anonymous and voluntary. The link to it was sent via email and distributed on social networks, following the snowball process. Before starting the survey, the study was briefly explained and then participants had to accept informed consent in order to begin responding. A non-probabilistic sample of 581 Spanish participants was obtained. The study was conducted in compliance with Spanish legislation (Ley Orgánica 3/2018, 5 December) and the code of ethics for research involving human subjects, as outlined by the Universitat de València Human Research Ethics Committee (ACGUV194/2006).

### Participants

The sample is composed of 581 participants. The average age is 30.38 years (*SD* = 13.30), ranging from 15 to 76 years. Most of the participants were women (67%), five people identified themselves with another gender, and three preferred not to answer. Almost half of the participants were students or students with temporary jobs (43.8%), 12.7% study and had a part-time job, 34.4% were full-time workers, 4.8% were unemployed people looking for a job, and 4.3% were unemployed without looking for a job or retired. The sample was mainly composed of single people (56.6%), with 39.1% married or living with a partner, 3.4% divorced, and 0.4% widowed. Finally, 63% had completed secondary education at most, and 37% had completed university or postgraduate studies. Regarding the vaccination process against Covid-19, 95.9% of the sample declared having received the coronavirus vaccine. In the case of unvaccinated people, the reasons stated were non-availability of vaccines in their region (*n* = 1), not meeting the criteria in the vaccination phase (*n* = 2), lack of interest in getting vaccinated (*n* = 11), lack of confidence in the results of vaccination (*n* = 6), fear of the side effects of the vaccine (n = 9), and other reasons (*n* = 8). The datasets analysed during the current study are available in the Harvard dataverse repository [32].

### Variables and instruments

The Vaccination Attitudes Examination (VAX) Scale is a brief 12-item questionnaire created to better understand general vaccination attitudes [21]. This scale evaluates four factors: trust of vaccine benefit, worries about unforeseen future effects, concerns about commercial profiteering, and preference for natural immunity. The response scale is a 5-point Likert-type scale, ranging from 1-Strongly disagree to 5-Strongly agree. Higher scores in the first factor indicate more trust, higher scores in factors two and three indicate more worry and concern, respectively, and higher scores in the fourth factor indicate more preference for natural immunity. The Spanish adaptation process of the VAX was conducted using the International Test Commission (ITC) criteria [28, 33]. The adaptation of the items of the scale was conducted using the translation–back translation method by two bilingual translators. The final version of the items of the scale in Spanish is shown in the Additional file 1.

Since the data was collected when vaccines for the coronavirus were already available, the concurrent validity has been studied using as a criterion whether the participants had been vaccinated or not against Covid-19. To assess it, a question with a dichotomous response (1. Yes, 0. No) was asked: “Have you been vaccinated against Covid-19?”.

### Data analysis

Confirmatory Factor Analysis (CFA) was used to study the factorial structure of the VAX. Two CFAs were calculated to test a single-factor and a four-related factor. The Maximum Likelihood Robust estimator (MLR) has been used. Although the response scale is ordinal, some studies suggest that MLR estimation can be used in confirmatory models when the data distribution is not normal and if the number of response categories for items is greater than four [34–36]. In this case, the variability in the parameter estimates is relatively small and MLR offers less biased standard error estimates as well as good estimates of the correlations between the factors [30, 31]. The reference values were 0.90 for the Comparative Fit Index (CFI), and a maximum cut-off of 0.08 for the Root-Mean-Square Error of Approximation (RMSEA) and for the Standard Root-Mean-Square residual (SRMR), to consider them as indicative of good fit model [31, 37, 38]. The factor measurement reliability was evaluated with the Composite Reliability index (CR) [39], which is identical to ω coefficient [29] because the standardized factor loadings have been used. Then, the Average Variance Extracted (AVE) was calculated to estimate the proportion of variance explained by each factor. Values equal to or greater than 0.70 for CR, and values equal to or greater than 0.50 for AVE are considered good [30]. For the model that best fit the data, the corrected item-total polyserial correlations for the items have been calculated, as indicators of corrected homogeneity indices for items with ordinal response scales [31].

Likewise, the measurement invariance according to gender and educational level has been studied for the best model, evaluated by calculating three nested invariance models that impose successive restrictions: configural, metric and scalar. Age invariance has not been calculated because almost 70% of the sample were people aged 30 or younger, and it did not make sense to form groups. To study invariance by gender, only two groups have been considered: men and women. To study the invariance by educational level, two groups have been formed: people who have completed secondary education at most (63%), and people with university or postgraduate studies completed. To assess the degree of invariance among the models, the following cut-off points in the increase of the indices have been considered: a change of 0.010 or greater in CFI along with a change of 0.015 or greater in RMSEA, or a change of 0.030 or greater in SRMR would indicate that there is no invariance [40]. Finally, to study the concurrent validity of the scale, a structural equation model has been specified considering the best model for the VAX scale as predictor of vaccination. Since the outcome variable is dichotomous (Vaccination yes or no), the odds ratio of the logistic regression, were also obtained. Furthermore, this validity model offers the estimation of the location parameter for the dichotomous variable (the parameter for the Rasch model). This parameter reports the minimum level of the trait from which a person is more likely to be vaccinated.

CFA, corrected item-total polyserial correlations, measurement invariance, and concurrent validity analyses were carried out with Mplus 8.8 [41], and for the description of the sociodemographic variables and statistics for the items of the VAX scale, IBM SPSS 23 was used.

## Results

### Confirmatory factor analyses and reliability

In Table 1 are shown the descriptive data of the VAX scale items and the item-total corrected polyserial correlations, that showed very good values and were statistically significant, ranging from 0.579 to 0.826.

**Table 1:** Statistics and corrected item-total polyserial correlations for the items of the Vaccine Attitudes Examination Scale

	Mean	SD	Skewness	Kurtosis	Item-total corrected polyserial correlations	Standard error for the item-total corrected polyserial correlations
Item 1	3.92	0.94	− 0.97	1.06	0.808	0.011
Item 2	4.28	0.83	− 1.33	2.30	0.671	0.021
Item 3	3.90	0.90	− 0.90	1.03	0.826	0.011
Item 4	3.89	0.81	− 0.68	0.54	0.579	0.024
Item 5	3.10	0.85	0.13	0.50	0.598	0.023
Item 6	3.22	1.10	− 0.15	− 0.72	0.588	0.022
Item 7	2.84	1.20	0.33	− 0.87	0.617	0.022
Item 8	2.39	1.11	0.61	− 0.26	0.730	0.016
Item 9	1.92	0.93	1.07	1.02	0.685	0.015
Item 10	2.86	1.00	0.00	− 0.10	0.627	0.017
Item 11	2.54	1.02	0.30	− 0.36	0.765	0.013
Item 12	2.38	1.01	0.48	− 0.17	0.707	0.015

**Fig. 1:**
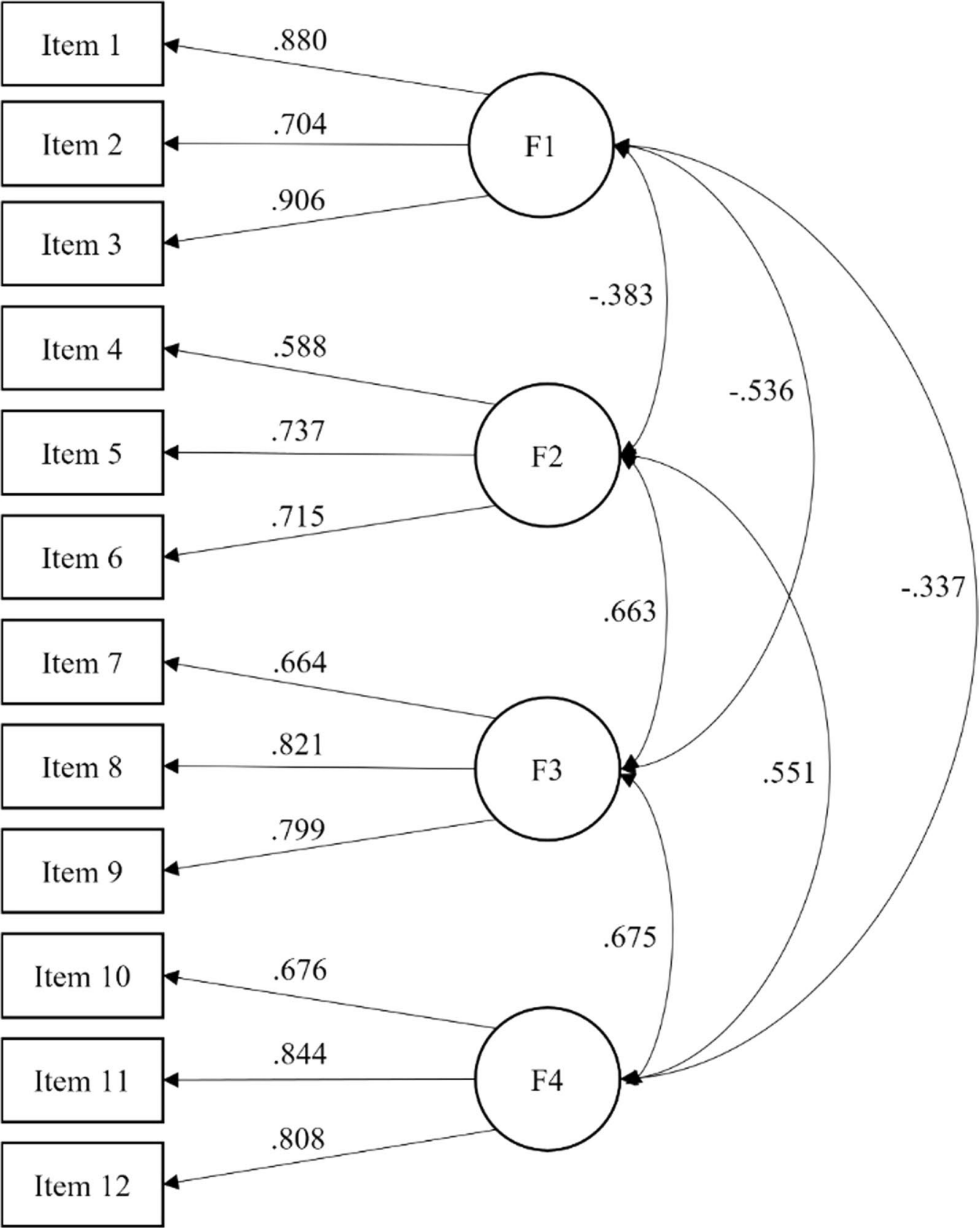
Standardized coefficients for the four-related factor model of the Vaccination Attitudes Examination Scale. *Note* F1 = Trust of vaccine benefit; F2 = Worries over unforeseen future effects; F3 = Concerns about commercial profiteering; F4 = Preference for natural immunity. All factor loadings and correlations among factors were statistically significant (*p* < 0.001)

Two CFA models have been tested to confirm the structure of the VAX in a Spanish sample. χ^2^ showed that the one-dimensional model was clearly inappropriate (χ^2^ (54) = 1,059.42, *p* < 0.001), as well as the other fit indices: CFI = 0.599, RMSEA = 0.179, RMSEA 90% CI = [0.170 − 0.189], and SRMR = 0.103. The four-related factor model showed very good fit regardless of the value of χ^2^ (χ^2^ (48) = 156.87, *p* < 0.001), with CFI = 0.957, RMSEA = 0.062, RMSEA 90% CI = [0.052, 0.073], and SRMR = 0.043. All factor loadings were statistically significant (*p* < 0.001) and above 0.580. Likewise, all the correlations among the factors were statistically significant (*p* < 0.001) and in the expected sense (see Fig. 1).

The Composite Reliability index (CR) was good for all the factors: F1 (CR = 0.818), F2 (CR = 0.717), F3 (CR = 0.790) and F4 (CR = 0.802). The Average Variance Extracted (AVE) was good for F1 (AVE = 0.697), F3 (AVE = 0.564) and F4 (AVE = 0.607), but slightly low for F2 (AVE = 0.467).

### Measurement invariance

In Table 2 are shown the results for the measurement invariance models by gender and by educational level. A good fit of the model can be observed in the four groups (men, women, up to higher education, with university studies), especially for men.

**Table 2:** Measurement invariance by gender and by educational level models, and goodness-of-fit indices

	χ^2^	*df*	Δχ^2^	Δ*df*	CFI	RMSEA	SRMR	ΔCFI	ΔRMSEA	ΔSRMR
*Gender*										
Men	64.45	48			0.980	0.048	0.044			
Women	136.61*	48			0.949	0.069	0.050			
Configural	200.98*	96	–	–	0.959	0.062	0.048	–	–	–
Metric	203.69*	104	10.6	8	0.961	0.058	0.053	0.002	− 0.004	0.005
Scalar	230.02*	112	20.0	8	0.946	0.061	0.057	− **0.015**	0.003	0.004
*Educational level*										
Up to higher education	116.88*	48			0.958	0.063	0.053			
With university studies	93.04*	48			0.950	0.066	0.046			
Configural	210.28*	96	–	–	0.955	0.064	0.050	–	–	–
Metric	217.48*	104	7.2	8	0.955	0.061	0.057	0	− 0.003	0.007
Scalar	233.89*	112	16.41	8	0.952	0.061	0.060	− 0.003	0	0.003

The change value that exceeds the invariance recommendations has been bolded

Reference group for Gender: Men. Reference group for Educational Level: Up to higher education

Δχ^2^ = chi-square change; Δdf = degrees of freedom change; CFI = Comparative Fit Index; RMSEA = Root-Mean-Square Error of Approximation; SRMR = Standardized-Root-Mean Square Residual; ΔCFI = CFI change; ΔRMSEA = RMSEA change; ΔSRMR = SRMR change. The change value that exceeds the invariance recommendations has been bolded. *p < 0.001

Regarding the measurement invariance models, the scalar invariance model for gender presents a change in CFI of the scaled metric model above the established limit. However, following Cheung and Rensvold (2002), a change of 0.010 or greater in CFI must appear together with a change of 0.015 or greater in RMSEA, in order to consider that there is no invariance, or there must be a change of 0.030 or greater in SRMR, which is not the case here either. Therefore, since the excessive change only occurred in CFI, it can be considered that the results showed scalar invariance, and the estimated latent means by gender and by educational level could be compared. After fixing latent mean values to zero for men, no differences for gender were observed in any of the factors: F1) b = 0.003, z = 0.037, *p* = 0.970; F2) b = 0.020, z = 0.387, *p* = 0.699; F3) b = − 0.053, z = − 0.659, *p* = 0.510; F4) b = − 0.041, z = − 0.575, *p* = 0.565).

The results of the invariance model by educational level showed strong invariance. After fixing latent mean values to zero for the “up to higher education” group, it was found that only the first factor (trust of vaccine benefit) showed non-significant differences (b = − 0.143, z = − 1.925, p = 0.054). However, people with university studies showed significantly higher means in F2 “concern about unforeseen future effects” (b = 0.125, z = 2.443, p = 0.015), F3 “concern about commercial effects and speculation” (b = 0.332, z = 4.669, *p* < 0.001) and F4 “preference for natural immunity” (b = 0.155, z = 2.431, *p* = 0.015).

### Concurrent validity

**Fig. 2:**
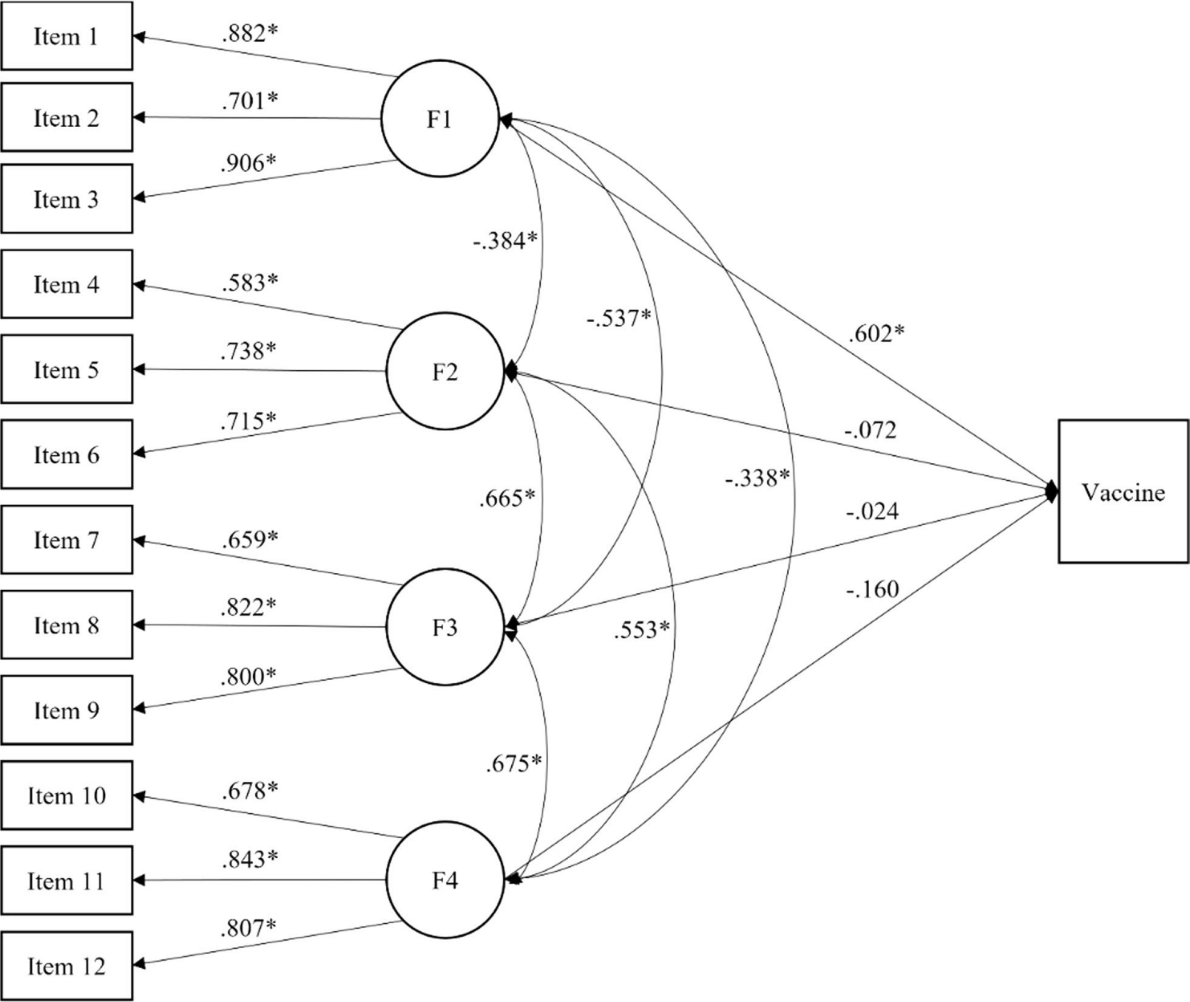
Standardized coefficients for the validity model of the Vaccination Attitudes Examination Scale. Reference group for Vaccine: Yes. *Note* F1 = Trust of vaccine benefit; F2 = Worries over unforeseen future effects; F3 = Concerns about commercial profiteering; F4 = Preference for natural immunity. *p < 0.001

In Fig. 2 is shown the validity model considering the four-related factor structural model as a predictor of been vaccinated. The results showed that the first factor “Trust of vaccine benefit”, was good predictor of being vaccinated. The first factor was a positive predictor of vaccination (*p* < 0.001). As the sign of the coefficient is positive (λ = 0.602), and the reference group is 1 (being vaccinated), this result indicates that the higher the score in this factor, the greater the probability of being vaccinated against Covid. The coefficients of the other two factors were not statistically significant. The odds ratio for the four factors are 6.777 (*SE* = 2.511), 0.669 (*SE* = 0.812), 1.185 (*SE* = 0.665), and 0.535 (*SE* = 0.344), respectively. On the other hand, the estimated value of *b* for the Vaccine variable is − 1.858 (*p* < 0.001). This means that, from a low level on the trait, people are more likely to be vaccinated than not to do so.

## Discussion

The objective of this study has been to adapt the Vaccination Attitudes Examination (VAX) scale in a Spanish sample, and to study its psychometric properties as well as the measurement invariance by gender and educational level. The results obtained in the CFAs report a very good fit of the model of four related factors in the present sample, as in the previous studies carried out with the VAX. Likewise, the corrected homogeneity indices and the Composite Reliability index report good reliability indicators for both the items and the subscales.

It should be noted that the value of the Average Variance Extracted is slightly lower than the cut-off point established in the second factor (worries over unforeseen future effects of the vaccine). This result could indicate that this factor may have less weight when explaining or predicting whether a person could be vaccinated or not, depending on the score obtained on it. Likewise, the existence of measurement invariance by gender and educational level has been verified, which indicates that it is possible to make comparisons between the latent scores in the factors for these groups.

The results indicate that, in this sample, there are no statistically significant differences in any of the VAX factors between men and women. However, there are differences depending on the level of studies in three factors: F2 “concern about unforeseen future effects”, F3 “concern about commercial effects and speculation” and F4 “preference for natural immunity”. Specifically, people with completed university studies present higher estimated latent scores in the three factors. However, there are no differences between both groups in Factor 1 (trust of vaccine benefit). These results may be in line with those found in some studies. A review of the literature found studies in China, Lebanon, Israel, Bangladesh, and the United States in which higher educational level had been identified as associated with vaccine hesitancy [42]. However, in another study conducted with data from 24 countries, no reliable relationship was found between educational level and vaccine hesitancy [43], although most of the sample were people with high educational levels. In the European Commission survey on vaccine confidence in countries of the European Union and the United Kingdom [44], it was found that people with primary education were more hesitant about vaccines only in four countries: Finland, Poland, Romania, and the UK. In general, it can be seen that there is little evidence showing an important influence of educational level on attitudes towards vaccines, and that when this information exists it may be different depending on the country. Some studies indicate that, more than educational level, individual cognitive styles and emotions are the ones that most influence reluctance towards vaccines [45].

On the other hand, the results of the validity model clearly indicate that the first factor of the VAX (trust of vaccine benefit) is the only significant predictor of whether a person decides to get vaccinated or not, while the other three factors are not relevant for predicting vaccination in this sample. Furthermore, only people with fairly low levels of being vaccinated or not (b = − 1.858), decide not to be vaccinated. In other words, only people who show high levels of mistrust in vaccines will decide not to get vaccinated. These results make sense if we take into account that more than 95% of the sample report having received the coronavirus vaccine. Perhaps for this reason, only the first factor predicts vaccination. In other words, despite showing a certain mistrust of the vaccine itself, of governments and pharmaceutical companies, and a certain preference for natural immunity, the vast majority of people in this sample have decided to get vaccinated. This is probably because, despite possible doubts or distrust of the vaccines developed against the coronavirus, the confidence in the vaccines is much higher.

### Limitations

Among the limitations of this study is the sample, since it is a non-probabilistic sample, and therefore, not representative of the population. Even so, the percentage of people in this sample who have been vaccinated is practically the same as that of Spain on the dates on which the sample was collected [46]. Likewise, it would be appropriate to get answers of people of different ages, since almost 70% of the sample is made up of people aged 30 or less. In this case, it would be convenient to study the measurement invariance by age.

### Future studies

It would be very interesting to carry out cross-cultural studies with the VAX to check which factors predict whether or not to be vaccinated depending on the country, since in the European Union report [44] it has been observed that Eastern European countries are the ones with the least confidence in vaccines. Measurement invariance studies between countries could inform whether the data are comparable between countries and knowing the reasons why people decide not to vaccinate or not to vaccinate their children can help governments and health authorities carry out campaigns in favour of vaccination, aimed at different types of population based on gender, age or educational level.

## Conclusion

The Vaccine Examination Attitudes (VAX) scale has shown adequate psychometric properties in Spain. Its structure of four related factors and its concurrent validity to predict whether people have been vaccinated or not are confirmed. Likewise, this scale presents measurement invariance by gender and educational level. It is confirmed that it is a very useful instrument to evaluate attitudes towards vaccines in Spain, which would allow obtaining more information about attitudes towards specific vaccines in the event of possible new pandemics, as well as to alleviate the slight rise in the anti-vaccine movement in Spain.

## Supplementary Information

**Additional file 1**. Items of the Vaccine Attitudes Examination (VAX) scale in Spanish.

## Data Availability

The datasets analysed during the current study are available in the Harvard dataverse repository (https://doi.org/10.7910/DVN/KZ66ES).

## Abbreviations

VAX: Vaccination attitudes examination scale
HIV: Acquired immunodeficiency virus
EFA: Exploratory factor analysis
CFA: Confirmatory factor analysis
MLR: Maximum likelihood robust estimation
CFI: Comparative fit index
RMSEA: Root-mean-square error of approximation
SRMR: Standardized root-mean-square residual
CR: Composite reliability index
AVE: Average variance extracted
