# Supplementary material for: Psychometric properties and measurement invariance of the Vaccination Attitudes Examination Scale (VAX) in a Spanish sample
Source: BMC Psychol. 2022 Sep 19;10:221. doi: 10.1186/s40359-022-00929-y (PMC9484348; doi:10.1186/s40359-022-00929-y)
Supplement: Supplementary file 1 — Additional file 1. Items of the Vaccine Attitudes Examination (VAX) scale in Spanish. [file 40359_2022_929_MOESM1_ESM.docx]

Appendix. Items of the Vaccine Attitudes Examination (VAX) scale in Spanish.

1. Me siento seguro(a) después de ser vacunado
2. Puedo confiar en las vacunas para detener enfermedades infecciosas graves
3. Me siento protegido(a) después de vacunarme
4. Aunque la mayoría de las vacunas parecen seguras, puede haber problemas que aún no hemos descubierto
5. Las vacunas pueden causar problemas imprevistos en los niños
6. Me preocupan los efectos desconocidos de las vacunas en el futuro
7. Las vacunas generan mucho dinero para las compañías farmacéuticas, pero no hacen mucho para la gente común
8. Las autoridades promueven la vacunación para obtener beneficios económicos, no por la salud de las personas
9. Los programas de vacunación son una gran estafa
10. La inmunidad natural tiene una duración más larga que una vacuna
11. La exposición natural al virus y los gérmenes brinda protección más segura
12. Estar expuesto(a) a enfermedades de manera natural es más seguro para el sistema inmunológico que estar expuesto(a) a través de la vacunación

Factor 1: Confianza en el beneficio de las vacunas (ítems 1, 2 y 3)

Factor 2: Preocupación por efectos futuros imprevistos (ítems 4, 5 y 6)

Factor 3: Preocupación por los efectos comerciales y la especulación (ítems 7, 8 y 9)

Factor 4: Preferencia por la inmunidad natural (ítems 10, 11 y 12)
